# Urinary Bladder Matrix as a Guide Bone Regeneration Barrier Membrane for Inhibiting Cell Invasion and Promoting Bone Formation

**DOI:** 10.1021/acsomega.5c03267

**Published:** 2025-07-30

**Authors:** Jie Zhong, Zhaoxin Chen, Yangqian Gu, Yiwen Xu, Wenyue Cheng, Jing Dai, Yang Sun, Siqing Yao, Mengmeng Lu, Jian Zhang

**Affiliations:** a School of Health Science and Engineering, University of Shanghai for Science and Technology, Shanghai 200093, China; b Department of Colorectal Surgery, 12521 Changzheng Hospital, Naval Medical University, Shanghai 200003, China; c Department of Oral Implantology, 168284 The Affiliated Stomatological Hospital of Nanjing Medical University, Nanjing, Jiangsu 210029, China; d State Key Laboratory Cultivation Base of Research, Prevention and Treatment for Oral Diseases, Nanjing, Jiangsu 210029, China; e Jiangsu Province Engineering Research Center of Stomatological Translational Medicine, Nanjing, Jiangsu 210029, China

## Abstract

Natural resorbable collagen membranes are widely used in guided bone regeneration (GBR) in oral implantology and prosthodontics. However, the rapid degradation and inadequate mechanical properties raise concerns about potentially compromising the ultimate bone regeneration efficacy. In this study, we developed a novel GBR membrane by coating a urinary bladder matrix (UBM) onto small intestinal submucosa (SIS), a commonly used acellular material, to improve the cell-barrier and bone-regeneration performances. The results showed that the UBM-SIS membrane exhibited superior tensile strength, high compliance, and slower degradation rate compared to a commercial Bio-Gide membrane, thereby enhancing the support and maintenance properties. In vitro studies indicated enhanced osteogenic behavior and higher osteogenic cytokine expression in human bone marrow-derived mesenchymal stromal cells (hBMSCs) cultured with UBM-SIS extract. In canine mandibular defect models, we proved that the UBM layer effectively resisted fibroblast invasion in the early stage, thereby enhancing the bone meal duration and osteoblast growth. Microcomputed tomography analysis revealed the greater quantity and maturity of bone trabeculae using a UBM-SIS membrane, exhibiting enhanced bone formation at 12 weeks and trabecular maturity at 24 weeks. In conclusion, we proposed a modified resorbable GBR membrane that may improve clinical outcomes in prosthodontics.

## Graphical abstract

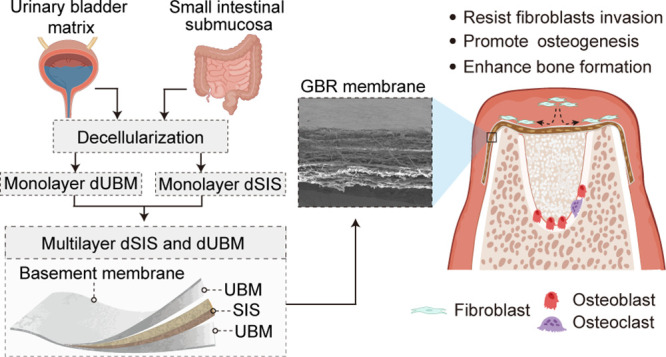

## 1 Introduction

Guided bone regeneration (GBR) is widely recognized as an important technique for alveolar bone reconstruction and treatment of peri-implant bone defects.^1–3^ Different cellular types exhibit varying migration rates during alveolar bone healing,^3–5^ while the GBR membrane acts as a barrier to preserve slower osteogenic cells and preventing faster epithelial or connective tissue infiltration.^1,6,7^ An ideal GBR membrane should be biocompatible, occlusive, easy to use, maintain space effectively, and bioactive.^3,8^

Currently available GBR barrier membranes include absorbable and nonabsorbable materials.^1,3^ Clinically prevalent absorbable collagen membranes have advantages in biodegradability, biocompatibility, tissue integration, promoted vascularization, and decreased membrane exposure risks.^3,8^ Nevertheless, they suffer problems in low mechanical strength and fast degradation, which lead to insufficient space maintenance and a limited duration of barrier function.^1,8,9^ Collagen membranes often lose strength soon after implantation,^1^ making it difficult to support space for osteogenesis, especially in large bone defects. The rapid degradation leads to scaffold structure collapse before the healing completed.^8,10^ It causes poor bone formation compared to nonabsorbable expended polytetrafluoroethylene (ePTFE) membranes, which maintain scaffold structure integrity better.^1^ However, nonabsorbable membranes often lead to complications and require removal, increasing costs and risks.^1^ Therefore, developing absorbable membranes with high mechanical properties and controlled biodegradation is critical for bone regeneration.

Decellularized materials, derived from the animal tissue extracellular matrix (ECM), which retain the ECM architecture and bioactivate components, can regulate cellular behaviors.^11^ The collagen within the ECM is organized into meticulously structured bundles of cross-linked fibers,^12–14^ endowing the decellularized materials with better mechanical strength.^11,13^ Acellular ECM scaffolds such as small intestine submucosa (SIS) and pericardium have been used as GBR barrier membranes.^9^ However, the porous structure also promotes cell invasion, leading to a quick degradation and barrier failure. In addition, the SIS exhibits potential inflammatory reactions, which might cause postoperative complications.^15,16^

The basement membrane (BM) is a highly specialized membrane located in most of the organs,^17–20^ which functions as a barrier between epithelial tissue and connective tissue and facilitate tissue isolation, connection, and transition.^19,21,22^ The BM network was constructure with Collagen IV and laminin, constituting the dense barrier structure.^17,18,23^ The urinary bladder matrix (UBM) is a biological scaffold composed of the basement membrane and lamina propria, which have been shown to maintain an intact BM layer during decellularized process.^24^ Additionally, UBM comprises of various collagen types, and bioactive components including proteoglycans, and growth factors, which can promote tissue regeneration.^12,25^ UBM has been suggested to promotes pro-healing M2 macrophages^12,25–28^ and has also been proved to attenuate osteoclast differentiation^29^ and reduce osteoarthritis.^30^ Clinically, UBM has been extensively utilized in the treatment of orthopedic wounds, including amputation wounds, and has been found to be an effective method for promoting composite regeneration.^31,32^ In vitro experiments demonstrated that cells cultured on UBM not only exhibited upregulated expression of osteogenic-related proteins, such as Runx2 and OPN, but also showed enhanced osteogenic activity.^33,34^ This is primarily attributed to the presence of various osteoinductive growth factors, including transforming growth factor-beta 1 (TGF-β1) and bone morphogenetic protein 4 (BMP4), in UBM.^35^ Therefore, UBM can serve as a typical material for GBR membranes owing to its excellent mechanical properties, superior biocompatibility, the ability to reduce inflammation, and osteogenic activity.

In this study, a novel UBM-SIS GBR membrane was developed by covering the multilayer SIS with UBM layers on the top surface to enhance the barrier efficiency and promote bone regeneration. The GBR membrane integrated with UBM has better mechanical properties and a slower degradation rate. The sandwich-structured design endows the membrane with a superior cell barrier effect and reduced inflammatory responses. Promoting-osteogenic potential of UBM-SIS was systematically evaluated through in vitro cell assays and an in vivo canine mandibular defect model. Results indicated that the UBM-SIS GBR membrane is a promising candidate material for clinical applications.

## 2 Materials and Methods

### 2.1 Preparation of Decellularized UBM and SIS Materials

Fresh porcine jejunum and urinary bladder were obtained from adult Yorkshire pigs and transported in ice–water. Then, the serous membrane, muscle layer, outer membrane, submucosa, and muscle layer of the bladder as well as the mucosal layer and outer muscle layer of the small intestine were mechanically removed. The decellularization process was based on a previous report.^36^ The decellularization level of decellularized UBM (dUBM) and decellularized SIS (dSIS) was determined by hematoxylin and eosin (HE) staining and DNA quantification. Samples were embedded in paraffin, cut into slices, and stained with hematoxylin and eosin. The amount of residual DNA was quantified using Quant-IT PicoGreen dsDNA Reagent and Kits (Thermo, USA) according to the manufacturer’s instructions.

### 2.2 Preparation of SIS and UBM-SIS GBR Membranes

All the decellularized GBR membranes were manufactured through layer-by-layer assembly of six decellularized matrix sheets on a flat platform with subsequent mechanical compaction. Nonlyophilized samples were prepared by the layer spread method. Individual decellularized sheets were flattened on a sterile platform, and each layer was carefully smoothed to eliminate interfacial air bubbles, uniformly compressed with a planar plate for 30 s, gently released, and then air-dried at room temperature (all steps performed <37 °C to preserve the ECM collagen structure). To enhance interlayer bonding, we optimized the process. Briefly, the assembled decellularized sheets were freeze-dried for 24 h while maintaining mechanically pressed. The UBM-SIS membrane was made by superimposing layers of UBM (upper and lower surfaces) and SIS (intermediate layers). UBM1-SIS and UBM2-SIS were prepared by sandwiching four or two layers of dSIS with one or two layers of dUBM on the top and bottom, respectively, for a total of 6 layers. The multilayer SIS membrane was prepared by 6 layers of dSIS. The preparation was carried out in a sterile workshop in ZhuoRuan Medical Technology (Suzhou) Co., Ltd. Membrane thickness was measured at 10 random points per sample using a micrometer, with three samples tested per batch across three production batches.

### 2.3 Micromorphology Characterization

The morphologies were characterized by using an SU-8010 field emission scanning electron microscope (SEM) (Hitachi, Japan) at 5 kV accelerating voltages. The samples were cut by using a knife in liquid nitrogen for cross-section views.

### 2.4 Mechanical Properties

Interfacial adhesion was characterized by a peel strength test using a CTM2050 material testing machine (Xieqiang Instrument Co., China). The sample (80 × 10 mm) was soaked in deionized water at 37 °C for 10 min, fixed on a glass plate, and underwent a 180° peel test. The stretch speed was set at 100 mm/min. For the uniaxial tensile test, the sample (30 × 10 mm) was hydrated and stretched to failure at a rate of 100 mm/min (gauge length was 20 mm). For the suture strength test, the sample (40 × 10 mm) was hydrated, and 4–0 surgical sutures were then inserted 5 mm from the edge of the membrane. The sutures were knotted at both ends, fixed to the tension gauge, and stretched at a rate of 100 mm/min until the suture point was torn, and the tension at the break point was recorded as suture strength. For the burst strength test, the round samples (65 mm in diameter) were hydrated and fixed on a ring fixture with an inner diameter of 45 mm. A spherical probe was passed through the sample at 300 mm/min.

The conformability of the membrane to bone defect boarders was indirectly assessed by a drapeability test,^37^ according to ISO 9073–9 (Textiles, Drape coefficient determination) and ISO 4604 (Textile glass, Determination of flexural stiffness). The sample (30 × 20 mm) was hydrated and then placed on a rectangular block with half of the length of the membrane hanging over the edge of the block. The conformability was graded according to the bending angle, 90° to 115° (complete), 115° to 140° (high), 140° to 165° (moderate), and 165° to 180° (minimal).

### 2.5 Degradation Properties

Degradation properties were evaluated using artificial saliva (Solarbio Life Sciences, Beijing, China). Briefly, the GBR membrane was cut into 10 × 30 mm pieces and dried at 37 °C for 4–6 h and weigh (W1). The samples were then immersed in artificial saliva (5 mg/mL) and incubated at 37 °C on a shaker. The samples were taken out at time points of 3, 7, 14, 21, 28, 42, and 56 d and then washed, dried, and weight (W2). Mass loss rate was calculated by the equation [(W1– W2)/W1] × 100%.

### 2.6 Cell Culture

L929 fibroblast cells (American Type Culture Collection, ATCC) were cultured in DMEM (Gibco, USA) with 10% fetal bovine serum (FBS, Cellmax, Australia) and 1% penicillin–streptomycin (P&S, Basamedia, China). Medium-equilibrated samples (1 × 1 cm) were seeded in 24-well plates. Cells were seeded onto the plates at a density of 1 × 10^4^ cells/well and incubated for 2 days (37 °C, 5% CO_2_). Cell adhesion was observed by SEM after fixation with 0.5% glutaraldehyde and dehydration through ethanol gradients. The in vitro cytotoxicity testing was performed following ISO 10993–5. Samples (6 cm^2^/mL) were extracted in DMEM with 10% FBS and 1% P&S at 37 °C for 24 h with shaking. L929 cells (1 × 10^4^ cells/well) were seeded in 96-well plates, cultured for 24 h (37 °C, 5% CO_2_), and then exposed to extracts for another 24 h. Cell viability was measured using an MTT kit (Adamas Life, Shanghai) with absorbance read at 570 nm.

Human bone marrow-derived mesenchymal stromal cells (hBMSCs) were isolated from jawbone specimens obtained through surgical procedures (approved by the Ethics Committee of the Nanjing Medical University (PJ2022–089–001)) and cultured in α-MEM containing 10% FBS and 1% P&S. Cells in passages 3 to 5 were used for the experiments, and the culture medium was refreshed every 3 days. All membranes were sterilized with ethylene oxide and cut into circles. Extracts were prepared by immersing the trimmed samples in α-MEM containing 10% FBS and 1% P&S, followed by incubation for 24 h at 37 °C on a shaker.

### 2.7 In Vitro ALP and ARS Staining

For alkaline phosphatase (ALP) staining, hBMSCs were seeded on 6-well culture plates at a density of 2 × 10^5^ cells/well and cultured with the previously described extracts. After 7 days, cells were washed with PBS three times and fixed with 4% paraformaldehyde for 30 min. ALP staining was performed using the BCIP/NBT Chromogenic Kit (Beyotime, China) per manufacturer’s instructions, with color development achieved through incubation for 12 h at 25 °C in darkness, and images were captured. To further assess calcium mineralization capacity, following 14 days of osteogenic induction with experimental extracts, cells were washed three times with PBS and fixed in 4% PFA for 1 h at room temperature. Mineralized matrix deposition was stained using 2% Alizarin Red S (ARS; Beyotime, China) for 15 min. After thorough PBS washing to remove unbound dye, calcium-rich mineralization nodules were visualized by light microscopy. For quantitative analysis, stained monolayers were destained with 10% cetylpyridinium chloride (CPC; MCE, USA) solution followed by 30 min shaking. The absorbance at 562 nm was measured by using a microplate reader.

### 2.8 Osteogenic Gene Expression Assays

Real-time quantitative polymerase chain reaction (RT-qPCR) was employed to assess the gene expression of various markers at different stages of osteoblast differentiation. hBMSCs were cultured in α-MEM medium supplemented with 10% FBS and 1% P&S for 4 and 7 days. Total RNA was extracted using TRIzol reagent (Vazyme, China). Subsequently, cDNA synthesis was performed using PrimeScript RT Master Mix (Takara, Japan). The reaction system was configured using the TB Green Premix Ex Taq (Takara, Japan) and referred to the instructions, and PCR amplification was performed using an ABI QuantStudio7 system. The target genes included RUNX2, ALP, COL1A1, and OCN, which were normalized to GAPDH, and the primer sequences are shown in Table 1. Gene expression levels were quantified by using the 2^–ΔΔCt^ method.

**Table 1:** Primer Sequences Used in RT-qPCR

gene	forward sequence (5′ to 3′)	forward sequence (5′ to 3′)
GAPDH	GGAGCGAGATCCCTCCAAAAT	GGCTGTTGTCATACTTCTCATGG
RUNX2	CCGCCTCAGTGATTTAGGGC	GGGTCTGTAATCTGACTCTGTCC
ALP	GTGAACCGCAACTGGTACTC	GAGCTGCGTAGCGATGTCC
COL1A1	GAGGGCCAAGACGAAGACATC	CAGATCACGTCATCGCACAAC
OCN	CACTCCTCGCCCTATTGGC	CCCTCCTGCTTGGACACAAAG

### 2.9 In Vivo Implantation in a Rat Model

Fifteen male Sprague–Dawley rats (240 ± 10 g) were purchased from Jihui Laboratory Animal Co., Ltd. (Shanghai) and randomly divided into three groups according to the observation time points at 1, 2, and 4 weeks. Muscle defects were made on both sides of each rat and randomly implanted with SIS, UBM1-SIS, and UBM2-SIS GBR membranes (*n* = 3 per group). Three samples were taken from each group for characterization. Rats were anesthetized with sodium pentobarbital (Beijing Kairuiji Biotechnology Co., China; 30 mg/kg, i.p.). A 5 cm long incision was made in the midsection of the abdomen to free the subcutis bilaterally. A section of 1.0 × 1.5 cm of the external and internal oblique muscles was excised along the lateral edge of the bilateral rectus abdominis without causing peritoneum damage. The membranes of the same size were hydrated in saline, covered the defect area, and then secured with interrupted 5–0 nonabsorbable sutures. The abdomen was closed with interrupted 3/0 absorbable sutures. The experimental protocol was approved by the Animal Care and Use Committee of the local government (Approval Number: P20200510067), and all procedures involving the animals followed the highest ethical standards, according to the Guide for the Care and Use of Laboratory Animals.

### 2.10 Mandibular Alveolar Bone Defect in a Canine Model

Twelve beagle dogs (11.5 ± 0.7 kg weight, Shanghai Xingang Experimental Animal Co., China) were randomly divided into three groups according to the observation time points at 4, 12, and 24 weeks (*n* = 4 per time point). Three defects were made for each dog, and Bio-Gide (Geistlich, Switzerland) or UBM-SIS GBR membranes were used to cover the defect site after bone powder filling, respectively. Bone powder and GBR membranes were not used in the blank group. After anesthetization with sodium pentobarbital (30 mg/kg, i.v.), the mandibular third and fourth premolars, the first and second molar teeth were extracted, the flap was sutured, and the normal saline washed and disinfected. After 8-week healing period, the dogs were again anesthetized with pentobarbital, the flap was opened, and 3 intrabone defects (10 × 5 mm, depth of 5 mm) were prepared on each side of the mandible. After filling with bone substitute granules (Bio-Oss, Geistlich, Switzerland), the defect area was covered with the GBR membranes and the flap was sutured without tension using 4–0 resorbable monofilament sutures.

### 2.11 Histological Evaluation

Rats were euthanized with sodium pentobarbital (200 mg/kg, ip) and confirmed death by the cessation of both respiration and heartbeat. Canines were euthanized with 10% potassium chloride solution (0.5 mL/kg (iv) following sodium pentobarbital (30 mg/kg, i.v.) anesthesia. For muscle defects in a rat model, the surgical area and the surrounding tissue were completely collected and fixed in 10% formalin for HE staining at 1, 2, and 4 weeks postsurgery. Briefly, the fixed sample was dehydrated through ethanol solutions, embedded in paraffin, sectioned, and stained by hematoxylin and eosin. The number of cells invading the membrane was counted using ImageJ by counting the number of cells within the membrane boundary in the field of view (800 × 600 μm) at 10× magnification. For mandibular alveolar bone defect in a canine model, the bone sample was fixed in 10% formalin, decalcified in 10% neutral ethylenediaminetetraacetic acid (EDTA) solution, embedded in paraffin, and sectioned. The slices were stained with HE and Masson’s stain to observe the degradation of the GBR membrane and the formation of new bone.

### 2.12 Microcomputed Tomography (Micro-CT) Evaluation

At 4, 12, and 24 weeks postsurgery, the surgical area and the surrounding bone tissue were taken out (the teeth, alveolar bone, and jaw were separated with a chainsaw, and the soft tissue was preserved) and fixed with paraformaldehyde solution for 48 h. A micro-CT scanner (SkyScan 1076, Bruker, Belgium) was used to measure new bone formation in the samples. The tissue volume (TV), bone volume (BV), bone surface (BS), bone volume fraction (BV/TV, %), bone surface fraction (BS/BV, %), trabecular thickness (Tb.Th), trabecular separation (Tb.Sp), and trabecular number (Tb.N) were analyzed and calculated (SkyScan CT-Analyzer).

### 2.13 Statistic Analysis

Data was expressed as mean and standard deviation (mean ± sd). One-way ANOVA test was used to compare data between three groups. An unpaired Student’s *t* test was used to compare data between any two experiment groups. *p* < 0.05 was used to determine statistical significance. *, *p* < 0.05; **, *p* < 0.01; ***, *p* < 0.001.

## 3 Results

### 3.1 dSIS and dUBM Materials

As shown in Figure 1a, after mechanical peeling and decellularization treatment, complete monolayer decellularized SIS (dSIS) and UBM (dUBM) membranes were prepared. HE staining results demonstrated complete nuclear removal, which was further verified by DNA content quantification, showing a significant decrease compared to native tissues (Figure 1b). The residual DNA content was below the established threshold standard of 50 ng/mg.^38^ SEM images revealed that dSIS had a rough texture and irregularly distributed pores on both mucosal and serosal sides. In contrast, the dUBM demonstrated an exceptionally smooth, flat, and compact surface on the BM side, which was attributed to preservation of the intact basement membrane (Figure 1c).

**Figure 1:**
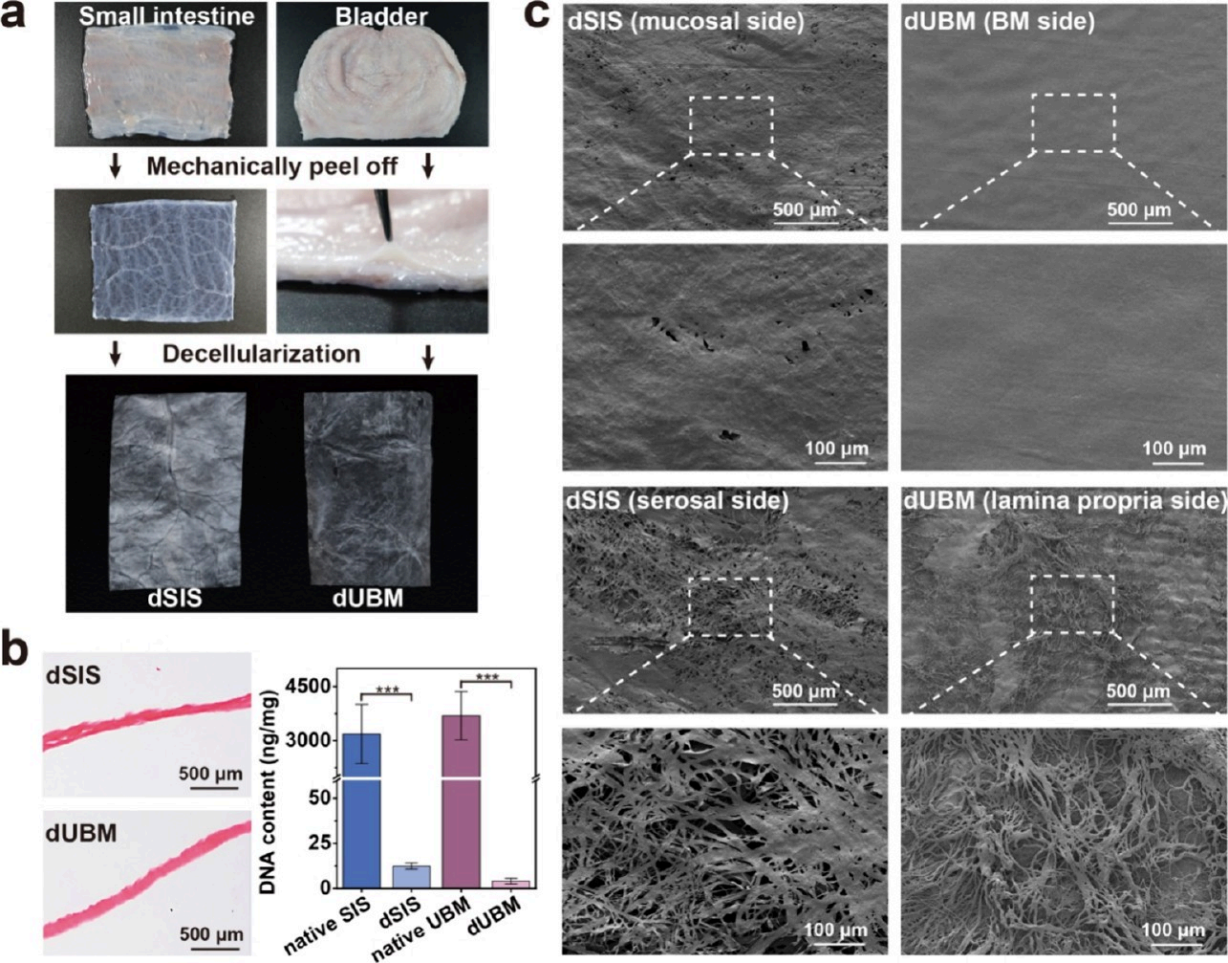
(a) Decellularization of the small intestine and bladder tissue for dSIS and dUBM preparation. (b) HE staining images of the dSIS and dUBM and DNA content quantification of the native and decellularized tissues (*n* = 3). ***, *p* < 0.001. (c) SEM images of the dSIS (mucosal side and serosal side) and dUBM materials (BM side and lamina propria side).

### 3.2 Structure and Mechanical Properties of the GBR Membranes

UBM with a smooth, dense, and nonporous surface was applied to the exterior of the SIS to develop a UBM-SIS GBR membrane with enhanced barrier functionality (Figure 2a). The flexible membrane could effectively cover the bone defect area, acting as a barrier to prevent soft tissue ingrowth while maintaining space for bone regeneration (Figure 2b). As shown in Figure 2c, L929 fibroblasts exhibited significantly enhanced adhesion on UBM-coated surfaces compared to SIS, and neither SIS nor UBM-SIS showed significant cytotoxicity.

**Figure 2:**
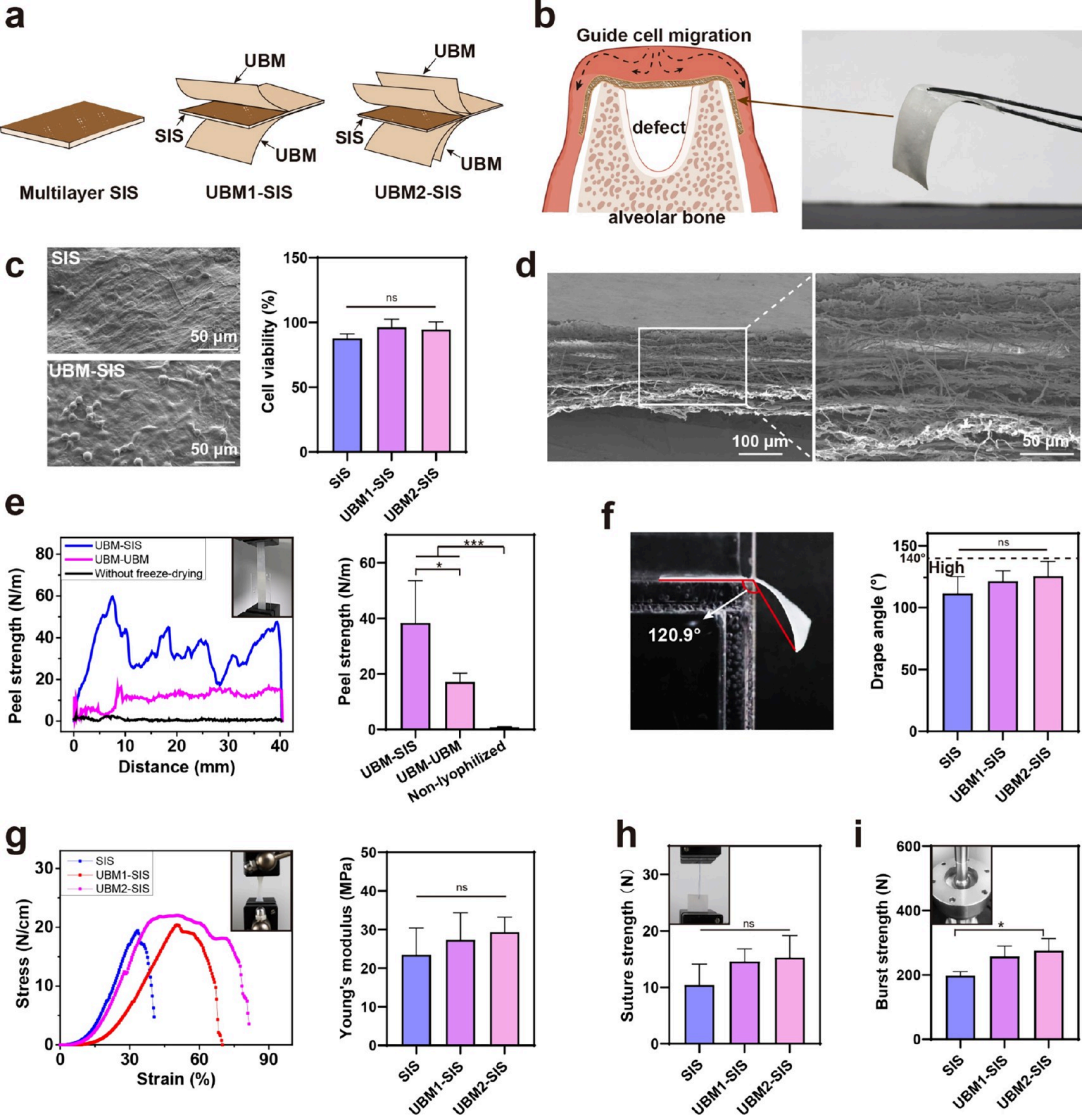
(a) Schematic diagram of the multilayer SIS membrane coated with the UBM. (b) Schematic diagram of the action principle of the GBR membrane. (c) The representative SEM image of L929 cells on the surface of SIS and UBM-SIS membranes and the cell viability assessment using an MTT kit (*n* = 3). (d) Micromorphology of the UBM-SIS membrane at cross section view. (e) The peel strength and static analysis at UBM-SIS, UBM-UBM, and nonlyophilized interlayer interfaces (*n* = 5). (f) Representative images and static analysis of the drapability test used to evaluate conformability and the drape angle of the SIS, UBM1-SIS, and UBM2-SIS membranes (*n* = 5). (g) Stress–strain curves of GBR membranes and static analysis of Young’s modulus (*n* = 5). (h) Suture strengths of the SIS, UBM1-SIS, and UBM2-SIS membranes (*n* = 5). (i) Burst strengths of SIS, UBM1-SIS, and UBM2-SIS membranes (*n* = 5). Not significant, ns, *p* > 0.05. *, *p* < 0.05; **, *p* < 0.01; ***, *p* < 0.001.

The mechanical properties of the SIS and UBM-SIS GBR membranes were further investigated. By controlling the consistency of raw materials, including pig breed, body weight, and harvest location, the thickness of different batches of SIS, UBM1-SIS, and UBM2-SIS membranes remained within the ranges of 207 ± 32, 203 ± 21, and 223 ± 31 μm, respectively. The UBM-SIS cross section morphology demonstrated the intricate entanglement of collagen fibers between the decellularized sheets (Figure 2d). Peel strength testing results revealed that nonfreeze-dried samples exhibited negligible binding force, and the peel strength between UBM and UBM was significantly weaker compared to that of UBM and SIS (Figure 2e). Both SIS and UBM-SIS became pliable and underwent deformation upon wetting (Figure 2f). The drape angle of SIS and UBM-SIS was less than 140° (Figure 2f), indicating high conformability, which means that the membranes could adapt to the surgical site and maintained in position by surrounding tissues. SIS, UBM1-SIS, and UBM2-SIS all exhibit greater tensile strength and modulus than the commercial Bio-Gide membrane (tensile strength: 7.4 ± 2.8 N/cm;^39^ Young’s modulus: 5.8 ± 0.7 MPa;^40^ Figure 2g). In the hydrated state, SIS, UBM1-SIS, and UBM2-SIS all exhibited high suture strength compared to the commercial collagen membranes,^41^ enabling them to be securely anchored to the surrounding tissue by the suture line. The burst strength of UBM2-SIS was superior to that of SIS, giving the membrane excellent occlusion.

### 3.3 Degradation Property

The degradation property of GRB was evaluated in artificial saliva. The SIS membrane exhibited a morphological alteration by the fourth week. In contrast, the UBM-SIS maintained its original shape by the eighth week, with only partial delamination observed in the UBM2-SIS during degradation (Figure 3a). The commercial collagen membrane (Bio-Gide) underwent substantial morphological alterations in the first week, resulting in weakened structural integrity. By the eighth week, the membrane was nearly imperceptible. The mass loss curve indicates that following immersion in artificial saliva for 1 week, the commercial membrane exhibited a mass loss of 84.4%, approaching complete degradation by the eighth week (Figure 3b). The mechanical properties of the GBR membranes after artificial saliva treatment were further investigated (Figure 3c). The commercial membrane Bio-Gide exhibited a complete loss of mechanical strength after 1 week. In contrast, SIS, UBM1-SIS, and UBM2-SIS maintained mechanical strength for 8 weeks, and the average residual tensile strengths were 0.25, 0.74, and 0.94 N/cm, respectively.

**Figure 3:**
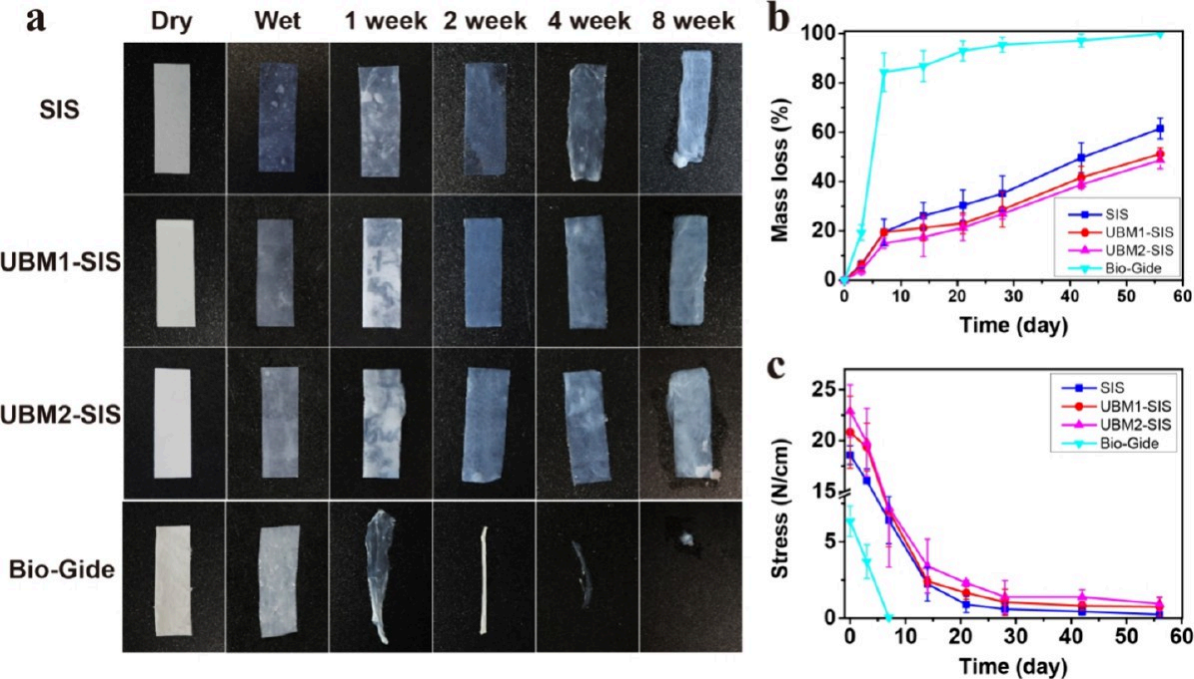
(a) Morphological changes of the membranes after degradation in artificial saliva at 1, 2, 4, and 8 weeks. (b) Mass loss curve of the membranes (*n* = 3). (c) Changes in tensile strength during degradation (*n* = 3).

### 3.4 Cell Barrier Effect of the GBR Membranes

To investigate the potential of the UBM-SIS layered membrane to function as a cellular guide and barrier, the SIS, UBM1-SIS, and UBM2-SIS membranes with identical layers were implanted in a rat abdominal wall defect. The results showed that at 1-week postsurgery, many cells had infiltrated all regions of the SIS membrane (Figure 4a, d). In contrast, there was negligible cellular invasion observed in the UBM1-SIS and UBM2-SIS membranes. Two weeks postsurgery, the SIS membrane group continued to display significant seroma, and part of membranes exhibited degradation-related breakage and disconnection, as well as substantial fibroblast invasion. UBM1-SIS and UBM2-SIS were also infiltrated by many inflammatory cells, but they preserved their morphological integrity and continuity (Figure 4b, d). By 4 weeks postsurgery, the SIS membrane was predominantly degraded with minimal residual fragments and persistent inflammation (Figure 4c, d). In contrast, the inflammatory response in the UBM1-SIS and UBM2-SIS membranes was markedly diminished and there was a significant reduction in cellular infiltration. All UBM-SIS membranes still maintained their structural integrity and continuity, enabling them to continuously and stably maintain the function of the GBR membranes.

**Figure 4:**
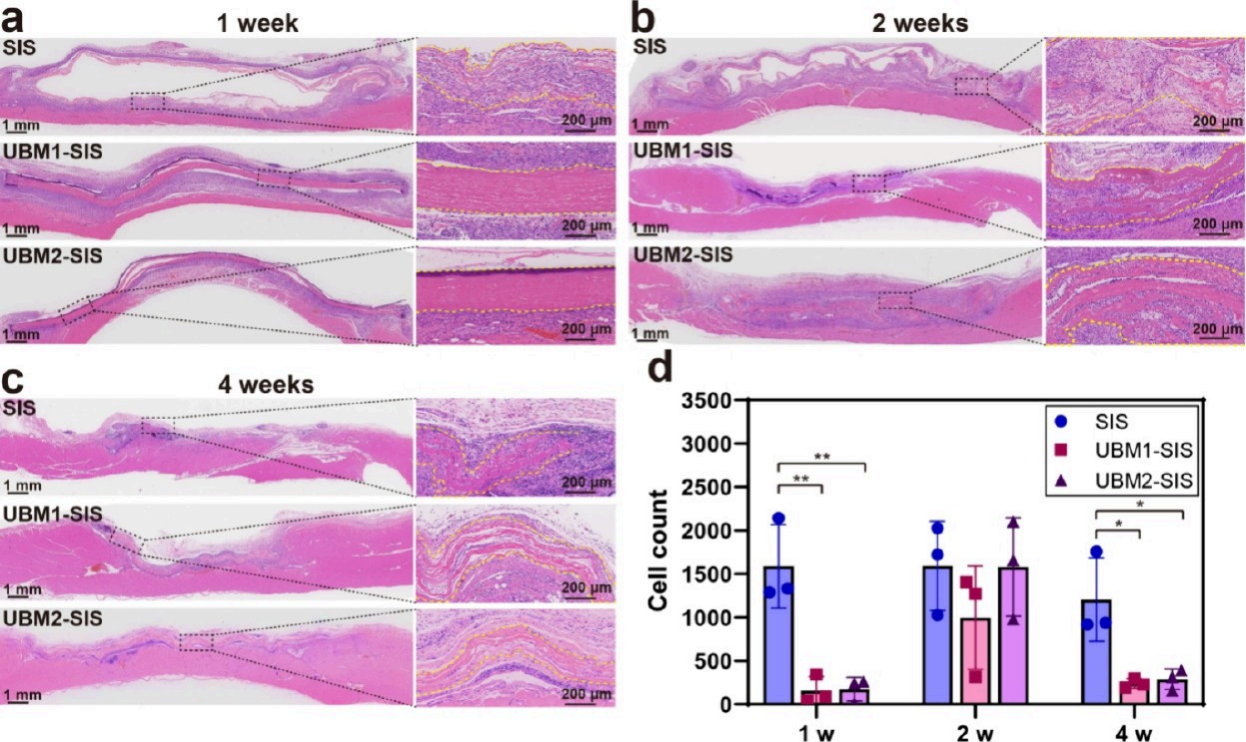
Effects of SIS, UBM1-SIS, and UBM2-SIS on guiding soft tissue cell migration in a rat model of abdominal wall muscle defect at (a) 1, (b) 2, and (c) 4 weeks after surgery. The yellow dotted lines indicate the boundary of the membrane. (d) Quantification of cells invading in the membrane (*n* = 3). *, *p* < 0.05; **, *p* < 0.01; ***, *p* < 0.001.

Above all, the introduction of UBM significantly increased its resistance to degradation and improved its function as a cellular guide and barrier compared with the SIS membrane. Also, no significant differences were observed between UBM2-SIS and UBM1-SIS. Given the potential delamination of the UBM2-SIS membrane during degradation, which may lead to structural instability, UBM1-SIS was selected for further investigation into the osteogenic properties of the UBM-SIS layered GBR membrane in vitro and in vivo.

### 3.5 Promoting-Osteogenesis Ability In Vitro

To evaluate the promoting-osteogenesis ability of the GRB membrane, hBMSCs were treated with GBR membrane extracts and stained by ALP and ARS, the biomarkers for early osteogenic differentiation and mineralized nodule formation, respectively. Results indicated that the GBR membranes significantly enhanced ALP expression compared to the blank group at 7 days (Figure 5a). Besides, the UBM-SIS group showed a higher optical density than Bio-Gide. After 14 days of culture, ARS staining revealed pronounced mineralized nodule formation in both GBR membrane groups, while UBM-SIS extracts further demonstrated stronger ARS intensity compared to Bio-Gide (Figure 5b).

**Figure 5:**
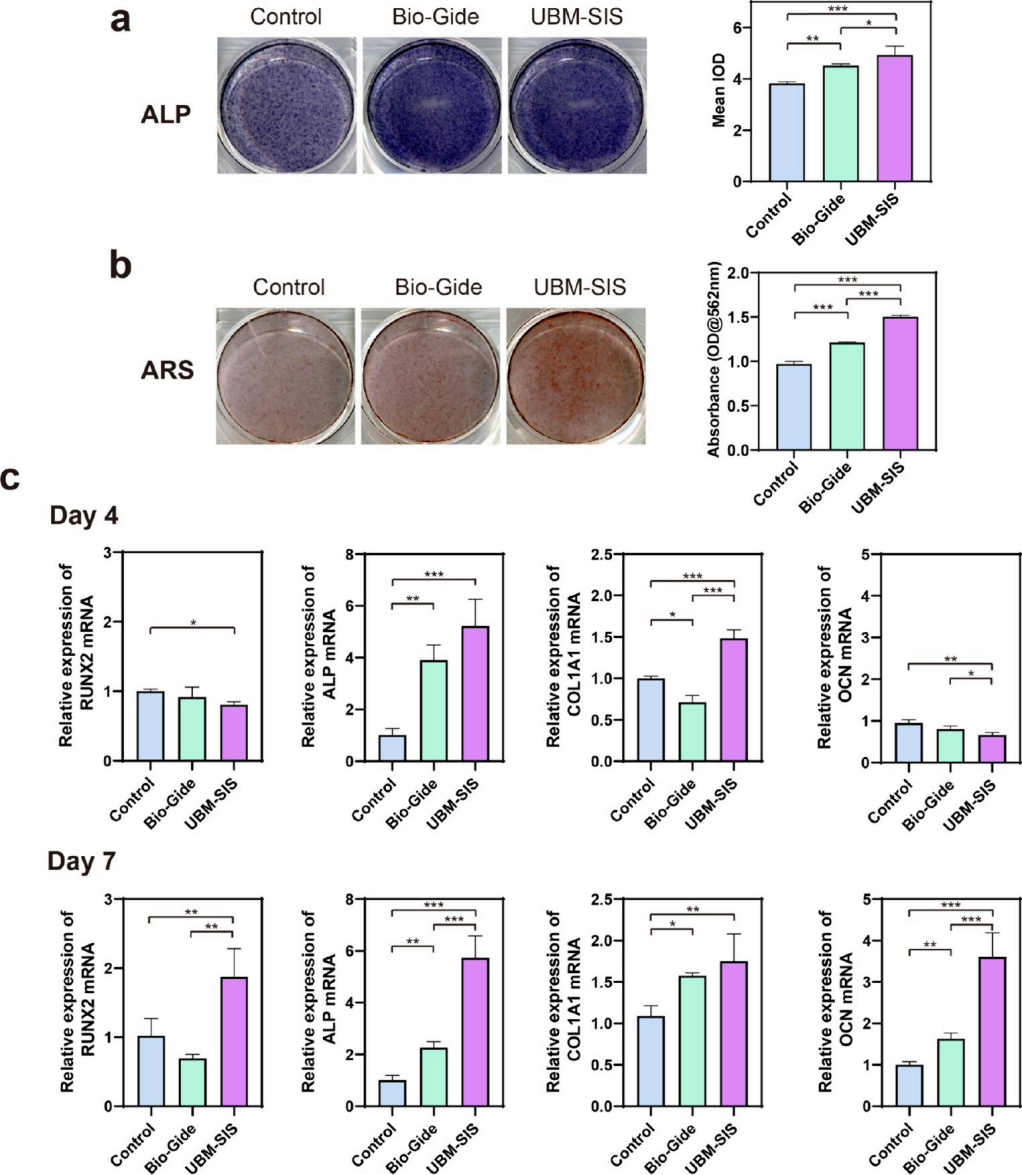
Representative images and quantification of (a) alkaline phosphatase (ALP, 7 days) and (b) Alizarin Red S (ARS, 14 days) staining of hBMSCs cells cultured in the extracts of GBR membranes. (c) PCR analysis of hBMSCs cultured in the extracts of GBR membranes for 4 and 7 days and the relative mRNA expression levels of osteogenic related genes RUNX-2, ALP, COL-1, and OCN in each group (*n* = 3). *, *p* < 0.05; **, *p* < 0.01; ***, *p* < 0.001.

To investigate the expression of osteogenic cytokines in hBMSCs, we evaluated the expression of runt-related transcription factor 2 (RUNX2), alkaline phosphatase (ALP), type I collagen (Col-l), and osteocalcin (OCN) as representative markers. As illustrated in Figure 5c, on day 4, the expression of the gene ALP, associated with early differentiation, was significantly elevated in the UBM-SIS group compared to the control and Bio-Gide groups. Additionally, the expression level of COL1A1, which is related to osteoblast adhesion and differentiation, was notably higher in the UBM-SIS group than in the other groups. By day 7, the expression levels of RUNX2, ALP, and OCN were markedly higher in the UBM-SIS group compared to both the control and Bio-Gide groups, with COL1A1 expression also significantly surpassing that of the control group. Furthermore, the expression of the late osteoblast marker, OCN in the UBM-SIS group on day 7 was significantly elevated compared to that on day 4, suggesting active osteogenesis. These findings indicated that osteoblasts cultured with the UBM-SIS extract exhibit enhanced activity and significantly improve hBMSC differentiation and bone mineralization.

### 3.6 Histological Evaluation in a Canine Mandibular Alveolar Bone Defect Model

The canine mandibular alveolar bone defect was treated by filling with bone substitute granules and subsequently covered with UBM-SIS and Bio-Gide membranes, as illustrated in Figure 6a. The typical HE staining image of the bone defect region is shown in Figure 6b. At 4 weeks postsurgery, the defect area in the blank group exhibited a large space. In the Bio-Gide and UBM-SIS groups, many small bone trabeculae were found in the defect area and no significant connective tissue invasion was observed (Figure 6b). At 12 weeks postsurgery, the blank group had a limited number of bone trabeculae and large areas of cavity. In the Bio-Gide and UBM-SIS groups, new bone formation was evident, characterized mainly by immature woven bone, with an increased bone trabecular connectivity density and occasional distribution of lamellar bone. By 24 weeks postsurgery, there were still large cavities in the defect area in the blank group. Meanwhile, the UBM-SIS and Bio-Gide groups demonstrated bone trabecular interconnection and an increase in lamellar bone within the defect area. These findings indicated that UBM-SIS effectively functions as a GBR membrane, promoting bone repair comparably to the commercial Bio-Gide.

**Figure 6:**
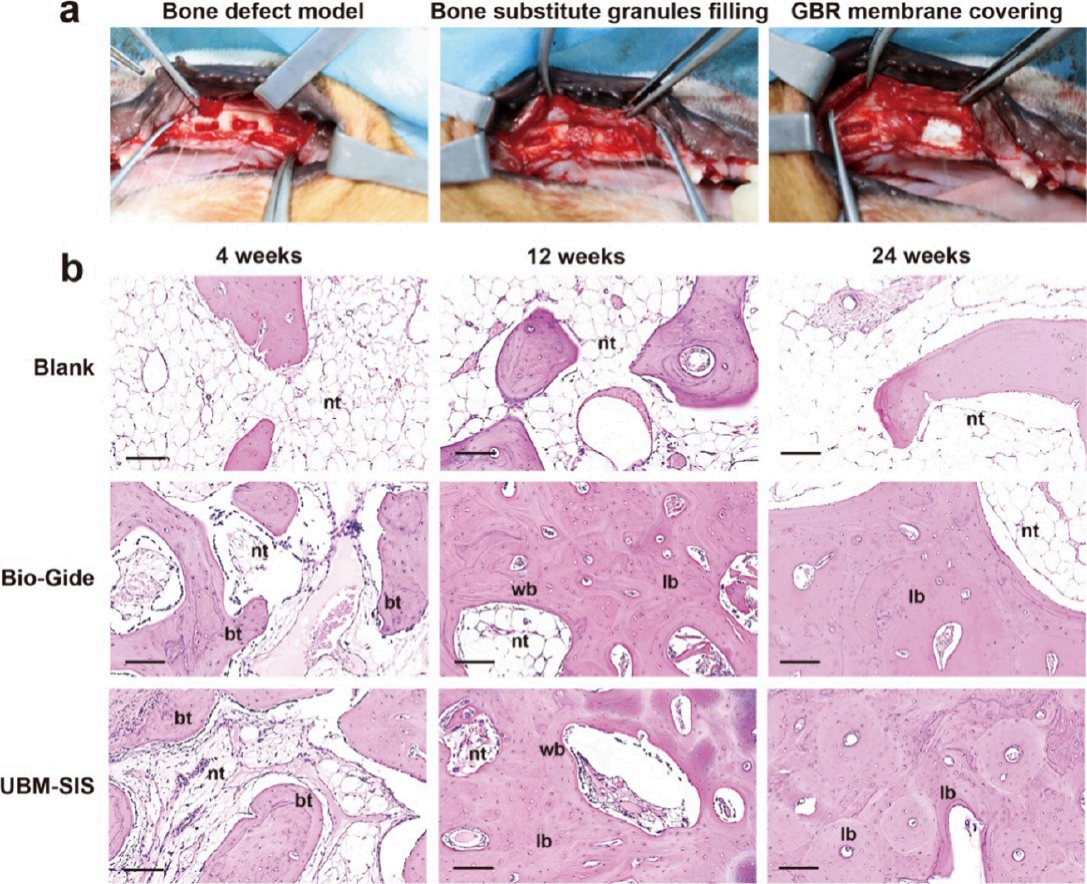
(a) Images of the bone defect model, bone substitute granules filling, and GBR membrane covering. (b) HE staining of the defect site in the canine mandibular alveolar bone defect model at 4, 12, and 24 weeks after surgery. Bone trabeculae (bt); woven bone (wb); lamellar bone (lb); nonmineralized tissue (nt). Scale bar: 100 μm.

The residue of the material and its integration with the tissue were evaluated by observing the junction of the GBR membrane with the bone. At 4 weeks postsurgery, the absence of a GBR membrane in the blank group allowed soft tissue to proliferate toward the bone, and a significant presence of osteoclasts at the interface impeded bone regeneration (Figure 7a, Figure S1). In contrast, both UBM-SIS and Bio-Gide groups exhibited a well-integrated GBR membrane with the surrounding tissue; a substantial presence of osteoblasts and a modest degree of bone regeneration were observed at the junction. Within the membrane, cellular infiltration was predominantly composed of inflammatory cells, which was accompanied by the development of some blood vessels. Twelve weeks postsurgery, the UBM-SIS group exhibited a significant reduction in inflammation. The residual membrane still effectively covered the bone defect area, and the residual amount of UBM-SIS was significantly higher than that of the Bio-Gide (Figure 7b, Figure S2). At 24 weeks postsurgery, the residual membrane was not detected in either the Bio-Gide or UBM-SIS groups. The histocompatibility of the GBR membrane was assessed based on the cell and tissue response scores of inflammatory cells, neovascularization, fibrosis, and fatty infiltrate. Also, the result showed that there were no significant differences between UBM-SIS group and Bio-Gide group (Figure S3).

**Figure 7:**
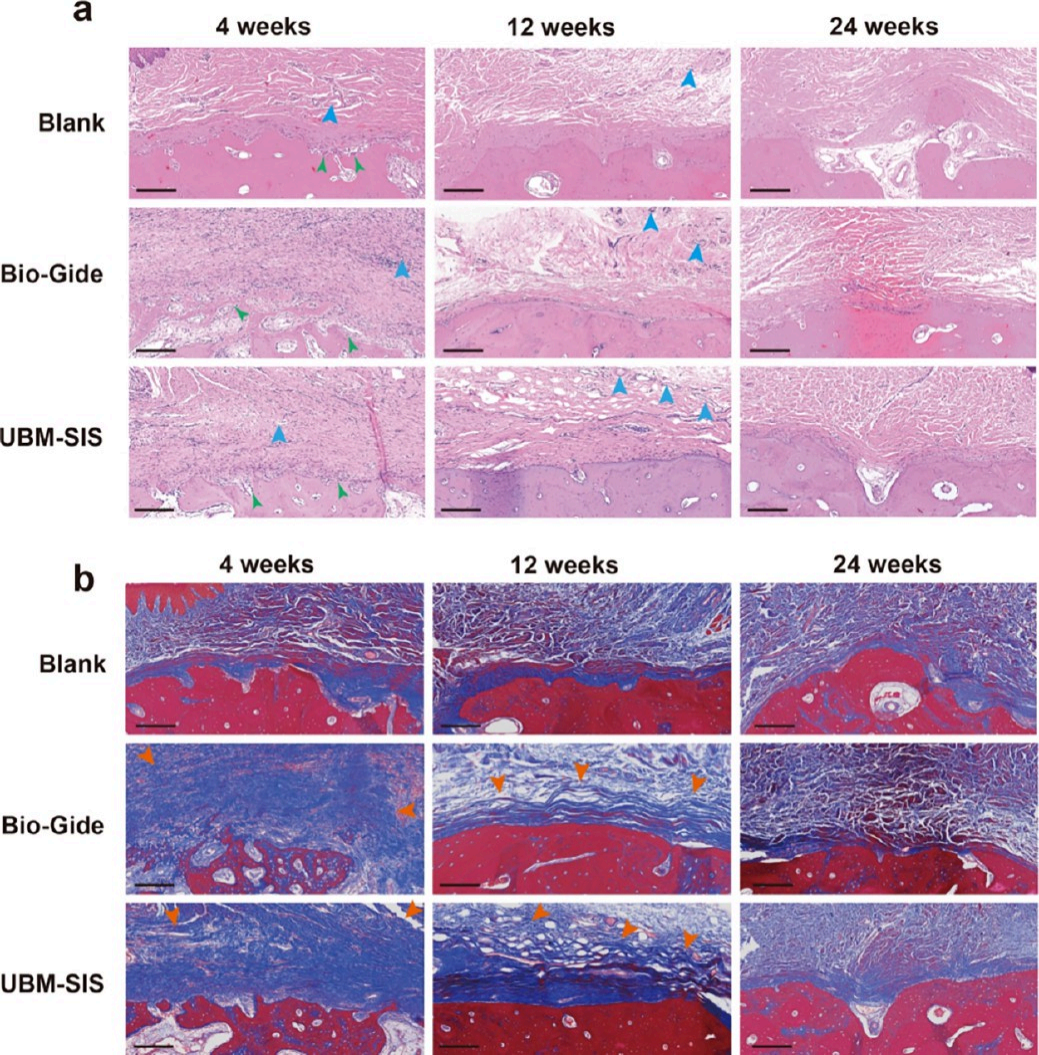
(a) HE and­(b) Masson’s staining at junctional regions in the canine mandibular alveolar bone defect model at 4, 12, and 24 weeks after surgery. Blood vessel (blue arrow); osteoblasts/osteoclasts (green arrow); residual membrane (orange arrow). Scale bar: 200 μm.

### 3.7 Micro-CT Evaluation

The typical three-dimensional computed tomography (CT) images of the alveolar bone at 4, 12, and 24 weeks postsurgery were presented Figure 8a. The bone defect exhibited a gradual healing process over time with the defect site progressively diminishing. Bone formation was quantitatively assessed using the five parameters: percent bone volume (BV/TV), bone surface-to-volume ratio (BS/BV), trabecular number (Tb.N), trabecular thickness (Tb.Th), and trabecular separation (Tb.Sp). Throughout the observation period, BV/TV showed a progressive increase, while BS/BV demonstrated a continuous decrease, indicating an overall enhancement in bone mass. Simultaneously, both Tb.N and Tb.Th increased, whereas Tb.Sp decreased over time, suggesting an advancement in bone structure maturation and stabilization of bone architecture. At 4, 12, and 24 weeks after surgery, BV/TV, Tb.N, and Tb.Th in the UBM-SIS and Bio-Gide groups were significantly higher than those in the blank group, and the BS/BV and Tb.Sp of the UBM-SIS group and Bio-Gide group were significantly lower than those of the blank group (Figure 8b, c). This indicates that the GBR membranes could isolate soft tissue to preserve bone formation space, thus significantly increasing bone increment and promoting bone regeneration. The BV/TV of the UBM-SIS group was significantly higher than that of the Bio-Gide group at 12 weeks after surgery, while the Tb.Sp at 24 weeks after surgery was significantly lower than that of the Bio-Gide group. These results suggest that UBM-SIS membranes have similar or even higher osteogenic effect than the commercial Bio-Gide GBR membrane.

**Figure 8:**
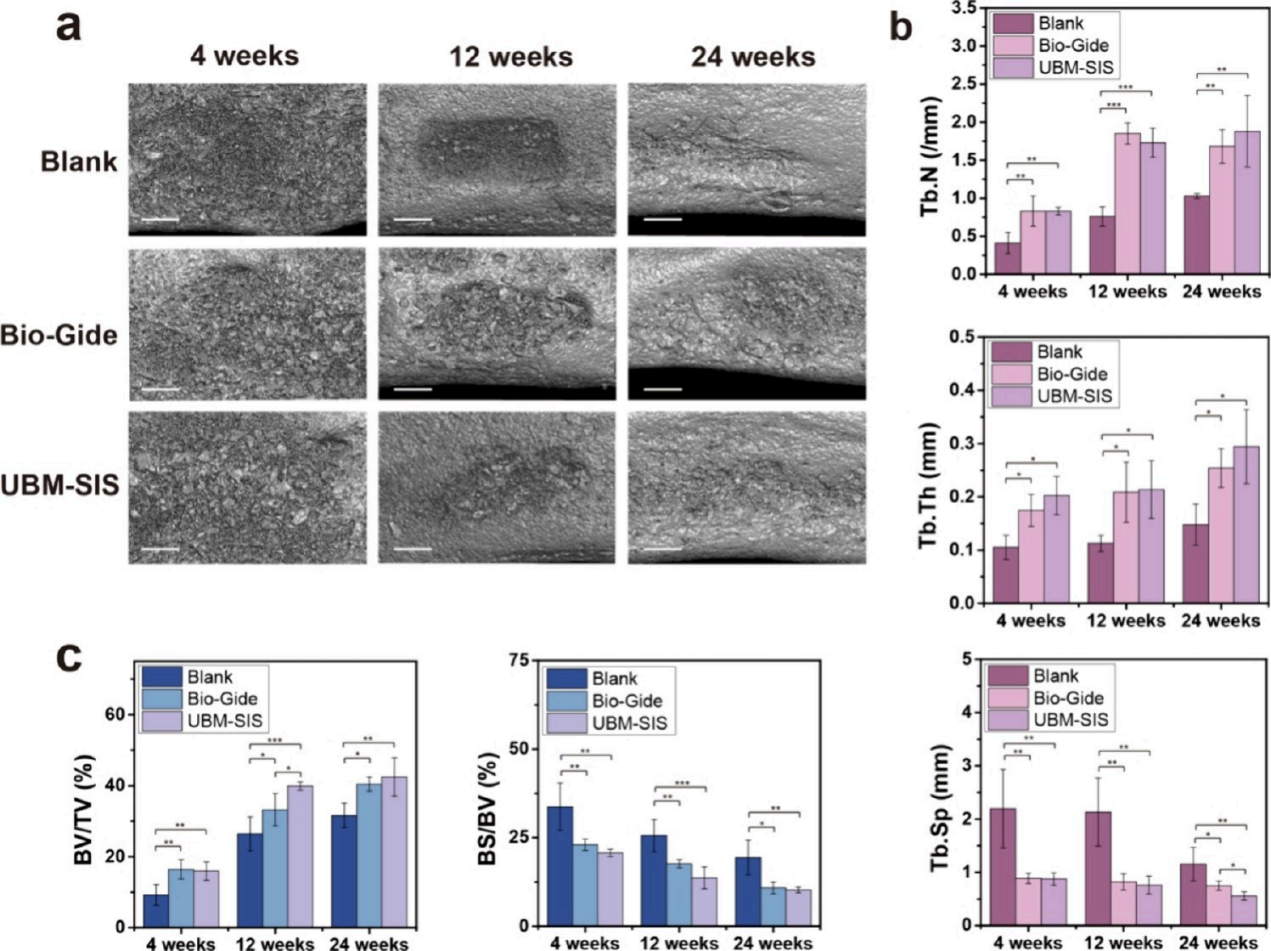
(a) Microcomputed tomography (micro-CT) images of bone repair in the canine mandibular alveolar bone defect model at 4, 12, and 24 weeks after surgery. Scale bar, 2.5 mm. (b) The number (Tb.N), thickness (Tb.Th), and separation (Tb.Sp) quantification of bone trabecula. (c) Quantification of the percent bone volume (BV/TV) and the bone surface/volume ratio (BS/BV) (*n* = 4). *, *p* < 0.05; **, *p* < 0.01; ***, *p* < 0.001.

## 4 Discussion

The optimal GBR membrane should exhibit biocompatibility, space-maintaining capability, ease of handling, occlusive function, and bioactivation potential. A critical requirement for GBR membranes is stable space maintenance, necessitating sufficient mechanical strength and a controlled degradation rate (at least 4–6 weeks).^3,9,42^ Conventional collagen membranes are typically derived from extensively purified animal collagen, a process that disrupts the natural fibrous architecture. As a result, these membranes often exhibit inadequate mechanical strength for space maintenance and undergo excessively rapid degradation.^8,10^ Although cross-linking is a prevalent strategy employed to improve strength and delay degradation, it may introduce cytotoxicity and inflammation.^8^ In contrast, UBM and SIS consist of naturally cross-linked collagen fiber bundles,^13,14^ endowing them with higher tensile strength and slower degradation than conventional collagen membranes while maintaining higher residual mechanical strength during degradation. Our physical compression method provided a simple yet controllable manufacturing approach that enabled direct scale-up for clinical production while preserving the native collagen fibrous architecture. Moreover, UBM-SIS exhibited a further reduced degradation rate and increased tensile strength than SIS alone, suggesting that UBM integration enhanced the structural stability for prolonged space maintenance. It should be noted that degradation tests were conducted in standardized artificial saliva to ensure reproducibility, although this static model cannot fully replicate the complex dynamic oral microenvironment.

Excessive rigidity can compromise tissue adaptation, whereas excessive softness may impair surgical handling.^3^ The UBM-SIS membrane balanced these properties, providing superior strength, while maintaining excellent pliability for surgical adaptation. Furthermore, its exceptional suture retention strength and burst resistance provided reliable occlusive function, significantly reducing the risks of membrane detachment or clinical failure. Notably, UBM-SIS interfaces exhibited strong binding strength, potentially due to the interlocking networks of collagen fibers between the interfaces under mechanically laminated.

Fibroblast exhibited invasive migration on porous SIS membrane, while growth on surface of basement membrane of UBM. Additionally, UBM appeared to induce an anti-inflammatory microenvironment,^12,25–28^ potentially suppressing inflammation-induced matrix metalloproteinase (MMP) secretion,^43,44^ and consequently slowing degradation. In vivo implantation results indicated delayed cellular infiltration, reduced inflammatory responses, and superior structural preservation of the UBM-SIS membrane compared to the SIS membrane. Our recent study suggests that UBM can mitigate characteristics of proinflammatory immune responses. Both UBM and fully decellularized SIS materials predominantly promote an M2 macrophage phenotype, while T cell infiltration is mainly composed of CD4^+^ helper T cells, indicating enhanced effects in tissue remodeling.^45^ The UBM surface provided physical guidance for cell migration while effectively blocking tissue invasion and reducing inflammation, thereby improving barrier efficacy and longevity of GBR membrane.

ECM comprises a complex of natural bioactive components, including diverse receptor ligands that mediate critical cellular processes.^11,12,25^ Particularly, UBM has demonstrated significant potential in promoting composite regeneration in amputation wound healing.^31,32^ The UBM-SIS membrane exhibited enhanced the promoting-osteogenic ability of hBMSCs compared to Bio-Gide, evidenced by ALP, ARS staining, and osteogenic cytokine expression analysis. Collagen peptides can promote osteogenic differentiation by activating the PI3K/Akt signaling pathway, thereby enhancing bone formation.^46,47^ In addition, UBM-SIS also contains abundant other bioactive ECM proteins, such as TGF-β1 and BMP4,^11,12,25,35^ which likely preserves critical signaling molecules and architectural cues that promote osteogenic commitment,^48,49^ though further investigation is necessary.

The bone augmentation results of the GBR membranes within a canine mandibular defect model indicated that both the UBM-SIS membrane and the commercial GBR membrane (Bio-Gide) effectively prevented the infiltration of soft tissue and facilitated bone trabeculae regeneration, which subsequently integrate and mature into lamellar bone. Micro-CT results showed that UBM-SIS significantly promoted bone repair, evaluated by the bone surface-to-volume ratio and trabecular thickness. Although the difference in trabecular thickness was not statistically significant, there was a significant reduction in trabecular separation, suggesting enhanced connectivity of bone trabeculae and a more mature bone architecture. Therefore, our results suggest that the UBM-SIS membrane we designed and prepared may exhibit superior bone regeneration effects compared to the commercial membrane, although further studies in more complex bone defects (e.g., infected or irregular) are needed to validate its potential advantages in such scenarios. Overall, the enhanced osteogenic potential of the UBM-SIS membrane compared to commercial collagen membranes suggests the GBR membrane is a promising candidate for guided bone regeneration applications.

## 5 Conclusions

The dense and nonporous natural barrier UBM was covered outside of the decellularized SIS to create the UBM-SIS GBR membrane. This layered membrane demonstrated superior mechanical support relative to both SIS and the commercial Bio-Gide membrane while maintaining high compliance that enhances clinical handling and patient comfort. Furthermore, the UBM-SIS membrane demonstrated superior stability and durability in spatial maintenance, with a significantly reduced degradation rate in artificial saliva compared to those of SIS and Bio-Gide. The incorporation of UBM also reduced inflammation and consequently enhanced the cell barrier effect, extending the duration of barrier maintenance. Additionally, compared to Bio-Gide, the UBM-SIS membrane significantly enhanced the osteogenic differentiation of hBMSCs. In a canine mandibular defect model, UBM-SIS effectively inhibited cellular infiltration into the bone defect and promoted new bone formation, and the bone volume, trabecular number, and maturity were significantly greater. Furthermore, both bone formation at 12 weeks and trabecular maturity at 24 weeks were significantly greater with UBM-SIS than with the commercially available Bio-Gide membrane. Consequently, UBM-SIS is a promising candidate material for clinical applications, and further research is required to explore its potential in complex defect repair and its immunomodulatory properties.

## Supplementary Material



## Notes

Enlarged HE staining image of junctional regions in the canine mandibular alveolar bone defect model, the thickness of the residual Bio-Gide and UBM-SIS membrane, and score of inflammatory cells, neovascularization, fibrosis, and fatty infiltrate in the regions surrounding the membrane (PDF)
