# Supplementary material for: Urinary Bladder Matrix as a Guide Bone Regeneration Barrier Membrane for Inhibiting Cell Invasion and Promoting Bone Formation
Source: ACS Omega. 2025 Jul 30;10(31):34831–43. doi: 10.1021/acsomega.5c03267 (PMC12355301; doi:10.1021/acsomega.5c03267)
Supplement: Supplementary file 1 [file ao5c03267_si_001.pdf]

Supporting information for

Urinary Bladder Matrix as A Guide Bone Regeneration Barrier Membrane for Inhibiting Cell Invasion and Promoting Bone Formation

Jie Zhong [a, b †], Zhaoxin Chen [a, b †], Yangqian Gu [c, d, e †], Yiwen Xu [b], Wenyue Cheng [b], Jing Dai [b], Yang Sun [b], Siqing Yao [c, d, e], Mengmeng Lu [c, d, e *], Jian Zhang [a, b *]

[a] School of Health Science and Engineering, University of Shanghai for Science and Technology, Shanghai 200093, China

[b] Department of Colorectal Surgery, Changzheng Hospital, Naval Medical University, Shanghai, 200003, China

[c] Department of Oral Implantology, The Affiliated Stomatological Hospital of Nanjing Medical University, Jiangsu 210029, China

[d] State Key Laboratory Cultivation Base of Research, Prevention and Treatment for Oral Diseases, Jiangsu 210029, China

[e] Jiangsu Province Engineering Research Center of Stomatological Translational Medicine, Jiangsu 210029, China

Correspondence:

Jian Zhang, Email: ZHJIAN_academic@outlook.com

Mengmeng Lu, E-mail: lumm@njmu.edu.cn

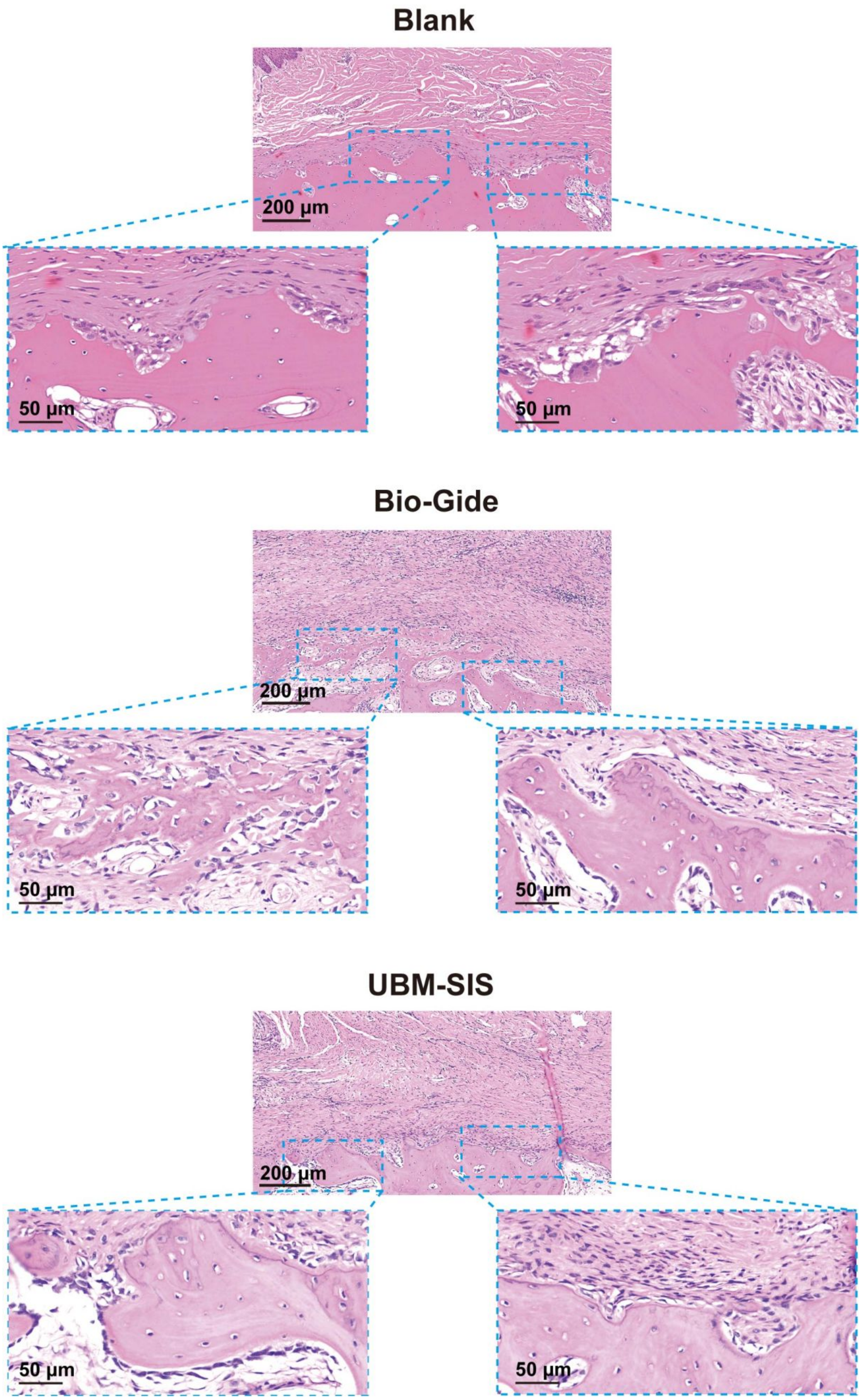

Figure S1. The enlarged HE staining image of junctional regions in canine mandibular alveolar bone defect model at 4 weeks after surgery.

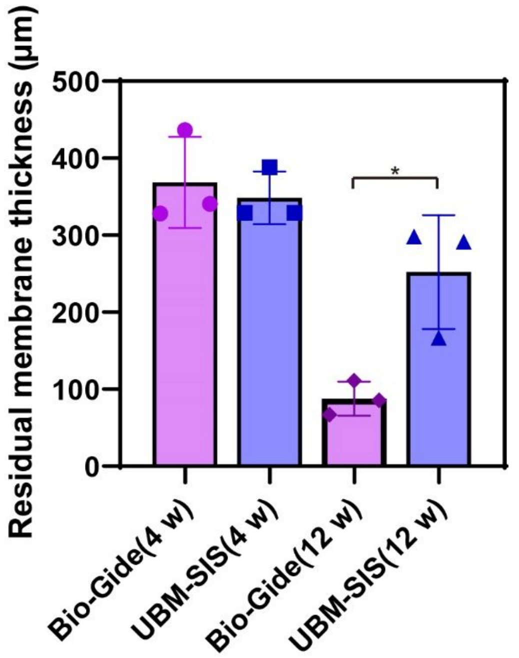

Figure S2. The thickness of the residual Bio-Gide and UBM-SIS membrane at 4 and 12 weeks. At 24 weeks, the membrane was difficult to detect.

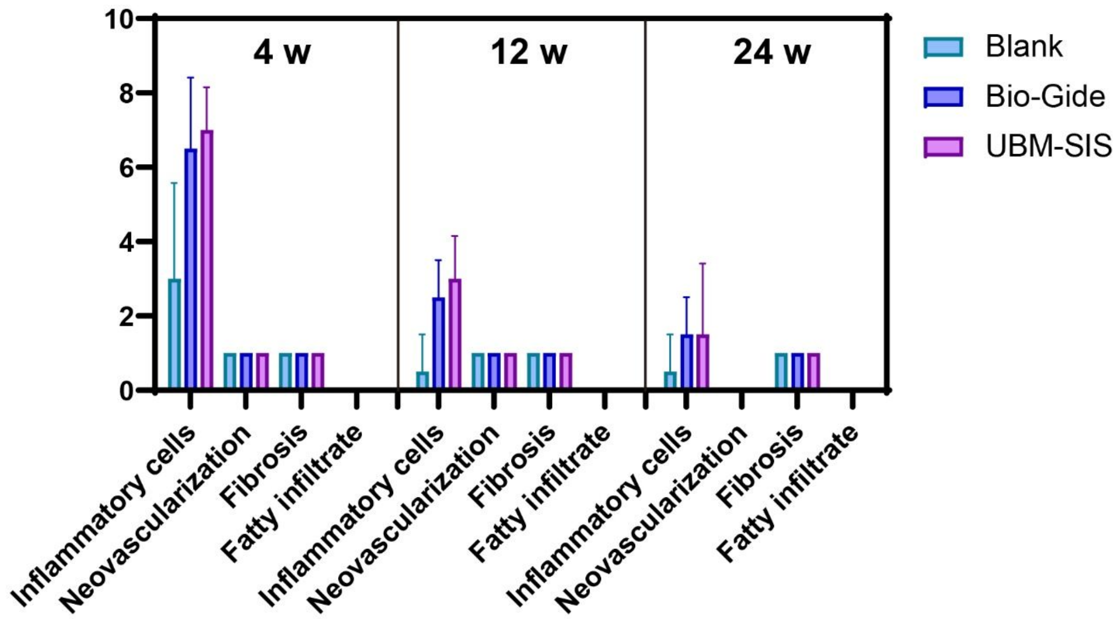

Figure S3. Score of inflammatory cells, neovascularization, fibrosis and fatty infiltrate in the regions surrounding the membrane at 4, 12 and 24 weeks.
